# Supplementary material for: Reconciling Mining with the Conservation of Cave Biodiversity: A Quantitative Baseline to Help Establish Conservation Priorities
Source: PLoS One. 2016 Dec 20;11(12):e0168348. doi: 10.1371/journal.pone.0168348 (PMC5173368; doi:10.1371/journal.pone.0168348)
Supplement: S1 Dataset — (ZIP) [file pone.0168348.s002.zip › Taxa/Serra Norte/SN_2013/N5S/Lista N5S-31.pdf]

CAVIDADE N5S-0031

Classe	Ordem	Fam/Outros	Gên/Outros	Espécie	S-U	U-S	Zon.
Annelida	Oligochaeta			sp.		1	E
Arachnida	Acari	(Oribatida)		sp.1		X	E
Arachnida	Acari	Laelapidae		sp.2	X		P
Arachnida	Amblypygi	Phrynidae	*Heterophrynus*	*longicornis*		1	E
Arachnida	Araneae	Caponiidae		jovem	X		E
Arachnida	Araneae	Ctenidae	*Nothroctenus*	*marschi*	4	3	E/P
Arachnida	Araneae	Drymusidae		jovem		X	P
Arachnida	Araneae	Filistatidae		jovem	X	X	E/P
Arachnida	Araneae	Ochyroceratidae		jovem	X		E
Arachnida	Araneae	Pholcidae	*Leptopholcus*	sp.1	X	X	E/P
Arachnida	Araneae	Pholcidae	*Mesabolivar*	*eberhard*	X	X	E/P
Arachnida	Araneae	Salticidae		jovem		X	E
Arachnida	Araneae	Scytodidae	*Scytodes*	jovem		1	P
Arachnida	Araneae	Segestriidae	*Ariadna*	sp.1	X		E
Arachnida	Araneae	Theridiosomatidae		jovem	X		E
Arachnida	Opiliones	Sclerosomatidae		jovem		X	E
Arachnida	Opiliones	Stygnidae	*Protimesius*	*gracilis*	2		E
Arachnida	Pseudoscorpiones	Chthoniidae	*Pseudochthonius*	sp.1	X		E
Arachnida	Pseudoscorpiones	Olpiidae		sp.1		X	E
Entognatha	Collembola	Entomobryidae		sp.3		X	E
Entognatha	Diplura	Campodeidae		sp.1	X		E
Insecta	Blattodea			jovem	1	1	E/P
Insecta	Diptera	Psychodidae	*Lutzomyia*	*longipalpis*		X	E
Insecta	Diptera	Psychodidae	*Pintomyia*	*gruta*		X	P
Insecta	Diptera	Psychodidae	*Sciopemyia*	*sordellii*	X		E
Insecta	Diptera	Sciaridae	*Bradysia*	sp.	X		E
Insecta	Diptera	Tipulidae	Tipulinae	sp.	X		E
Insecta	Heteroptera	Reduviidae	Reduviinae	jovem	1	1	E
Insecta	Heteroptera	Reduviidae	Schizopterinae	jovem		X	E
Insecta	Hymenoptera	Formicidae	*Camponotus*	sp.1	X		E
Insecta	Hymenoptera	Formicidae	*Crematogaster*	sp.3		X	E
Insecta	Hymenoptera	Formicidae	*Solenopsis*	sp.2		X	E
Insecta	Isoptera	Termitidae	*Labiotermes*	sp.	X		E
Insecta	Isoptera	Termitidae	*Nasutitermes*	sp.	X	X	E/P
Insecta	Lepidoptera	Hesperioidea		sp.	1		E
Insecta	Lepidoptera	Noctuoidea		sp.2	X	X	E/P
Insecta	Lepidoptera	Noctuoidea		sp.11		X	E
Insecta	Lepidoptera	Noctuoidea		sp.12	8	1	E
Insecta	Lepidoptera	Noctuoidea		sp.13	5		E
Insecta	Orthoptera	Phalangopsidae	*Paraclodes*	sp.	1	2	E
Insecta	Orthoptera	Phalangopsidae	*Phalangopsis*	sp.	18	8	E
Insecta	Psocoptera	Psyllipsocidae	*Psyllipsocus*	sp.1		X	E/P
Insecta	Psocoptera	Ptiloneuridae	*Ptiloneura*	sp.1		X	E
Malacostraca	Isopoda	Dubioniscidae		sp.1		X	E/P
Turbellaria	Tricladida			sp.	X		E
Mammalia	Chiroptera	Emballonuridae	*Peropteryx*	sp.		3	P
Reptilia	Squamata	Gekkonidae	*Thecadactylus*	sp.	1		E
